# Supplementary material for: End Sequence Analysis Toolkit (ESAT) expands the extractable information from single-cell RNA-seq data
Source: Genome Res. 2016 Oct;26(10):1397–410. doi: 10.1101/gr.207902.116 (PMC5052061; doi:10.1101/gr.207902.116)
Supplement: Supplemental Material [file supp_gr.207902.116_Supp_Materials.tar.gz › ESAT-esat_v0.1/doc/html/classumms_1_1esat_1_1_collapsed_gene_lengths.html]

ESAT: umms.esat.CollapsedGeneLengths Class Reference


ESAT


- Main Page
- Related Pages
- Packages
- Classes
- Files

- Class List
- Class Index
- Class Hierarchy
- Class Members

All Classes Namespaces Files Functions Variables Enumerator Pages

- umms
- esat
- CollapsedGeneLengths

Public Member Functions |
Static Public Member Functions |
List of all members

umms.esat.CollapsedGeneLengths Class Reference

Collaboration diagram for umms.esat.CollapsedGeneLengths:

[legend]

|  |  |
| --- | --- |
| Public Member Functions | |
|  | CollapsedGeneLengths (String[] args) throws IOException |
|  | |

|  |  |
| --- | --- |
| Static Public Member Functions | |
| static void | main (String[] args) throws IOException |
|  | |

## Constructor & Destructor Documentation

|  |  |  |  |  |  |
| --- | --- | --- | --- | --- | --- |
| umms.esat.CollapsedGeneLengths.CollapsedGeneLengths | ( | String[] | *args* | ) | throws IOException |

Here is the caller graph for this function:

## Member Function Documentation

|  |  |  |  |  |  |
| --- | --- | --- | --- | --- | --- |
| static void umms.esat.CollapsedGeneLengths.main | ( | String[] | *args* | ) | throws IOException |

static

Parameters
:   |  |  |
    | --- | --- |
    | args |  |

Exceptions
:   |  |  |
    | --- | --- |
    | IOException |  |

Here is the call graph for this function:

---

The documentation for this class was generated from the following file:

- java/umms/esat/CollapsedGeneLengths.java


---

Generated on Fri Aug 15 2014 15:11:15 for ESAT by  

 1.8.7
